# De novo *VPS16* missense variant causes infantile-onset dystonia with defective autophagic flux

**DOI:** 10.1172/jci.insight.207998

**Published:** 2026-06-30

**Authors:** Enrique Gonzalez Saez-Diez, Xutong Xue, Amy Tam, Hyo-Min Kim, Siofra Carty, Joshua Rong, Monica Ferrer-Socorro, Kathryn Yang, Darius Ebrahimi-Fakhari

**Affiliations:** 1 Movement Disorders Program, Department of Neurology, F.M. Kirby Neurobiology Center, Boston Children’s Hospital, Harvard Medical School, Boston, Massachusetts, USA.; 2 Medical Faculty, Heidelberg University, Heidelberg, Germany.

**Keywords:** Genetics, Neuroscience, Autophagy, Movement disorders, Neurodevelopment

## Abstract

A VPS16 gene variant causes movement disorder in an infant by blocking cellular waste clearance — confirmed in patient cells and potentially treatable with deep brain stimulation.

*VPS16* encodes a core structural subunit of the homotypic fusion and vacuole protein sorting (HOPS) complex, which orchestrates Rab7-mediated tethering and SNARE-dependent fusion of autophagosomes with lysosomes. Heterozygous loss-of-function (LOF) variants in *VPS16* cause autosomal-dominant DYT-*VPS16* dystonia (OMIM #619291), a recognizable syndrome of early-onset generalized dystonia driven by impaired endolysosomal trafficking and autophagic flux in striatal neurons (1). Over 80 patients have been reported, predominantly carrying truncating or splice-altering alleles; the pathogenicity of missense variants has remained uncertain because direct functional evidence in patient-derived cells has been lacking (2). Here, we describe a 2-year-old child with infantile-onset generalized dystonia due to a de novo *VPS16* missense variant, the youngest patient with DYT-*VPS16* reported to date, and demonstrate that this allele disrupts autophagic flux in patient fibroblasts, establishing haploinsufficiency as the disease mechanism. We further describe a previously undocumented structural neuroimaging correlate of this disorder.

A girl of Kenyan ancestry, born at term after an uncomplicated pregnancy with an unremarkable neonatal course, presented at age 9 months with failure to achieve rolling or independent sitting, axial hypotonia, and emerging generalized dystonia involving the craniocervical region, limbs, and trunk (Supplemental Video 1; supplemental material available online with this article; https://doi.org/10.1172/jci.insight.207998DS1). The diagnosis was delayed due to the atypical early onset, which fell outside the range previously described for DYT-*VPS16* (previous minimum ~2 years) (3). A dystonia gene panel, chromosomal microarray, and clinical exome sequencing with mitochondrial DNA analysis were non-diagnostic, and the variant was ultimately identified by research trio short-read genome sequencing. Brain MRI at age 2 demonstrated symmetric, caudate-predominant striatal volume loss with ex vacuo ventricular dilatation (Figure 1, A and B).

Genome sequencing identified a de novo heterozygous missense variant in *VPS16* (NM_022575.4): c.1396G>A, p.Ala466Thr. The variant was absent from gnomAD (PM2), arose de novo (PS2_VeryStrong), was predicted damaging by multiple in silico tools (CADD PHRED 29.2, REVEL 0.844, AlphaMissense 0.8917; PP3), and was concordant with the DYT-*VPS16* phenotype (PP4). Under ACMG/AMP criteria, the variant was classified as Likely Pathogenic. Structurally, Ala466, a highly conserved residue across species (Supplemental Figure 1A), maps to the interface between the N-terminal β-propeller and C-terminal α-solenoid domains of *VPS16*, a region critical for HOPS complex assembly and for positioning the Sec1/Munc18 module, comprising VPS16 and VPS33, to coordinate SNARE complex priming during autolysosome biogenesis (4). LOF variants in *VPS16* and additional HOPS-complex genes (*VPS11*, *VPS41*) are associated with early-onset progressive movement disorders (1, 5).

To determine whether p.Ala466Thr disrupts lysosomal integrity, we performed transmission electron microscopy (TEM) of patient-derived fibroblasts. Patient cells displayed markedly enlarged vacuolar structures morphologically consistent with stalled autolysosomes, in contrast with the compact endolysosomal compartments observed in age- and passage-matched control fibroblasts (Figure 1, C and D, and Supplemental Figure 1, B and C). This ultrastructural phenotype closely resembles that reported by Steel et al. (1) in fibroblasts from patients carrying LOF alleles, supporting equivalent functional disruption by this missense variant.

To quantify autophagic flux, we examined steady-state levels of p62/SQSTM1, an autophagy cargo receptor degraded upon productive autolysosome formation, and the autophagosome membrane marker LC3B by immunoblotting in patient and control fibroblasts (Figure 1E and Supporting Data Values file). Patient cells exhibited significantly elevated p62 protein (Figure 1F; *P* < 0.05, unpaired *t* test, *n* = 3 biological replicates) and an elevated LC3-II/LC3-I ratio (Figure 1G; *P* < 0.05). The concurrent accumulation of both markers is the cellular hallmark of a block in autophagic flux; whereas isolated LC3-II elevation can reflect increased autophagosome formation, co-elevation of p62, which is itself a substrate for autophagic degradation, indicates that cargo is not being cleared. Together with the TEM data, these results establish that p.Ala466Thr impairs the terminal step of autophagy, namely autolysosome maturation and cargo degradation, recapitulating the mechanism documented for LOF alleles of *VPS16*.

This case makes 4 contributions to the DYT-*VPS16* literature. First, it provides functional cellular evidence that a missense allele can cause haploinsufficiency-equivalent HOPS dysfunction, with direct implications for variant interpretation. Second, the caudate-predominant striatal atrophy on MRI represents a previously undescribed neuroimaging signature that, if confirmed in additional patients, could serve as a diagnostic biomarker and a structural readout for disease progression and therapeutic monitoring. This is potentially consistent with preferential autophagy-dependent vulnerability of caudate medium spiny neurons — a hypothesis that remains to be tested. Third, the disease onset in infancy extends the recognized clinical spectrum of DYT-*VPS16* into the first year of life, emphasizing that infantile-onset generalized dystonia of unknown cause warrants *VPS16* sequencing. Fourth, the patient’s Kenyan ancestry highlights a gap in the geographic representation of DYT-*VPS16* cohorts and underscores the importance of inclusive sequencing programs. Crucially, the confirmed molecular diagnosis enables access to deep brain stimulation (DBS) of the internal globus pallidus, for which a recent multicenter analysis demonstrated significant benefit (6). This justifies early referral for DBS evaluation in young patients with refractory DYT-*VPS16* dystonia, although prospective studies are needed (7).

## Conflict of interest

The authors have declared that no conflict of interest exists.

## Funding support

This work is the result of NIH funding, in whole or in part, and is subject to the NIH Public Access Policy. Through acceptance of this federal funding, the NIH has been given a right to make the work publicly available in PubMed Central.

- National Institute of Neurological Disorders and Stroke grants K08NS123552 and 1U54NS148312).
- Spastic Paraplegia Foundation.
- Dystonia Medical Research Foundation.
- Boston Children’s Hospital Translational Research Program.
- Boston Children’s Hospital TIDO Accelerator Award.
- Dystonia Medical Research Foundation fellowship (to MFS).

## Supplementary Material

Supplemental data

Unedited blot and gel images

Supplemental video 1

Supporting data values

## Figures and Tables

**Figure 1:**
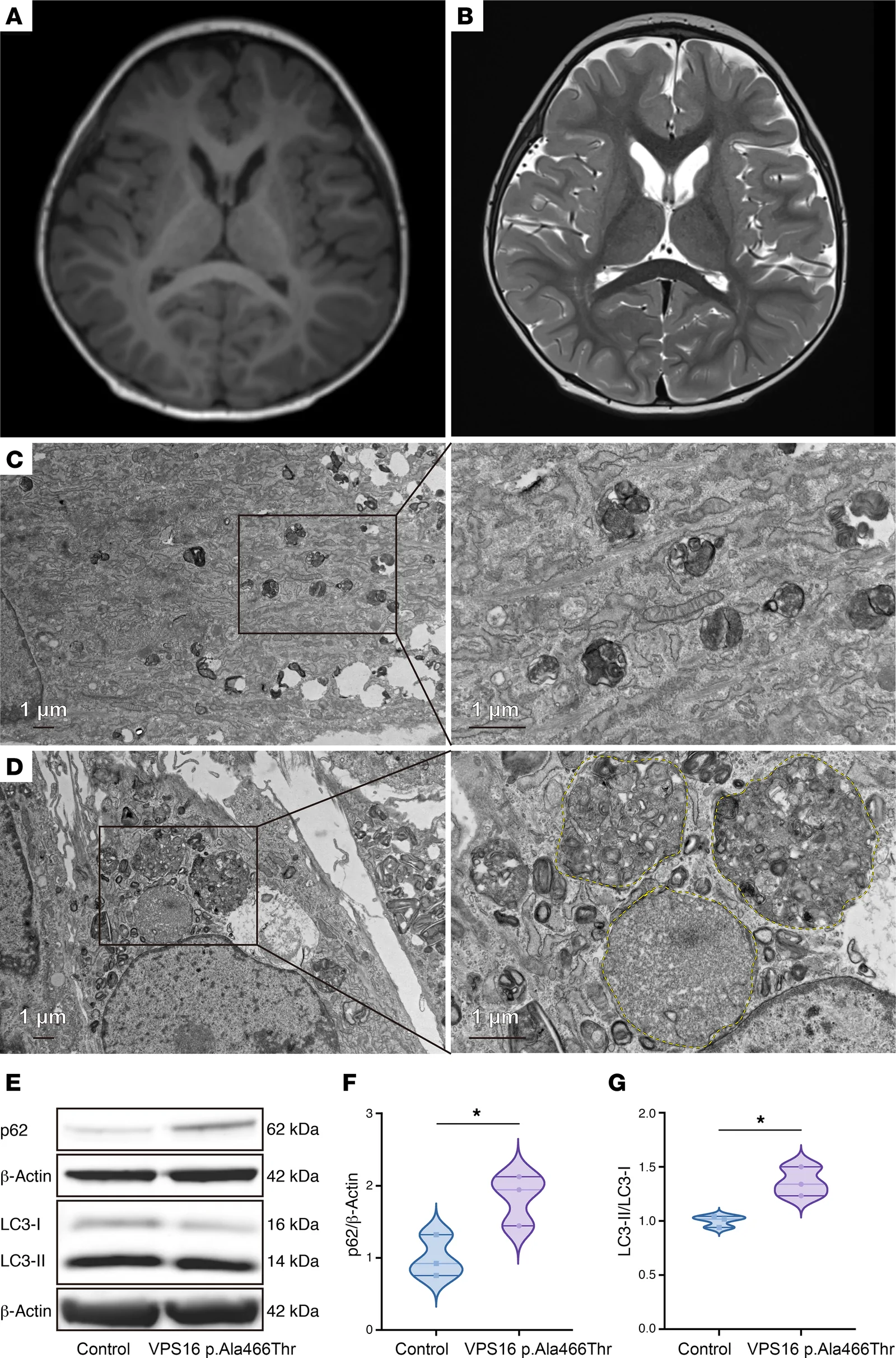
De novo *VPS16* missense variant p.Ala466Thr causes caudate atrophy and impaired autophagic flux in patient-derived fibroblasts. (**A** and **B**) Brain MRI demonstrating symmetric, caudate-predominant striatal volume loss with ex vacuo dilatation of the frontal horns (**A**, T1-weighted axial; **B**, T2-weighted axial). No T2 signal abnormality, mineralization, or putaminal involvement is present. (**C** and **D**) Transmission electron micrographs of control (**C**) and patient (**D**) skin fibroblasts. Yellow dashed circles in **D** mark enlarged vacuolar structures consistent with stalled autolysosomes, absent in control cells. Scale bars: 1 μm. (**E**) Representative immunoblots for p62/SQSTM1, LC3B (LC3-I and LC3-II), and β-actin (loading control) in control and patient fibroblasts. (**F**) Quantification of p62 protein levels normalized to β-actin (*n* = 3 biological replicates). *P < 0.05, unpaired *t* test. (**G**) Quantification of the LC3-II/LC3-I ratio (*n* = 3 biological replicates). *P < 0.05, unpaired *t* test. Data presented as violin plots with individual data points.

## Version 1 06/30/2026

In-Press Preview

## Version 2 08/24/2026

Electronic publication
