# Supplementary material for: De novo VPS16 missense variant causes infantile-onset dystonia with defective autophagic flux
Source: JCI Insight. 2026 Jun 30;11(16):e207998. doi: 10.1172/jci.insight.207998 (PMC13505382; doi:10.1172/jci.insight.207998)
Supplement: Supplementary file 1 [file jciinsight-11-207998-s019.pdf]

SUPPLEMENTAL MATERIAL

<u>Supplemental Methods</u>

Clinical Case History

The patient is a female infant of Kenyan ancestry, conceived by in-vitro fertilization to healthy, non-consanguineous parents (maternal age 47 years at delivery), and born at 38+4 weeks gestation by planned cesarean delivery following an uncomplicated pregnancy. The neonatal course was unremarkable, with appropriate feeding and weight gain through the first several months of life. Developmental concerns first emerged at 9 months when she had not achieved rolling or independent sitting. Initial evaluation at 10 months revealed head lag, intermittently increased lower limb tone, persistent hand fisting without voluntary grasp, and inability to sit unassisted. By 19 months, she presented with global developmental delay and increased tone. She was unable to sit or roll, wore ankle-foot orthoses, and could bear weight when held but struggled to propel a gait trainer. A modified barium swallow study demonstrated silent aspiration, requiring thickened feeds. Despite significant motor impairment, she remained socially engaged with appropriate eye contact, smiling, and early syllabic vocalizations ("mama," "dada," "baby"). Examination at 2 years revealed severe axial hypotonia with inability to hold her head upright, coupled with appendicular hypertonia and dystonic posturing of all four limbs (Supplemental Video 1). Hands were predominantly fisted with mild intermittent choreiform finger movements. Deep tendon reflexes were normal and symmetric without clonus, and plantar responses were down-going. No distinct dysmorphic features were noted, and there were no skeletal abnormalities or neurocutaneous stigmata. Hearing and vision were clinically intact.

Diagnostic Workup

Comprehensive metabolic evaluation was unremarkable, including plasma amino acids, acylcarnitine profile, lactate, pyruvate, and urine organic acids. Cerebrospinal fluid (CSF) analysis, including neurotransmitter metabolites, was normal. Nerve conduction studies were normal. Spine MRI was normal. Initial brain MRI at age 1 year was reported as normal; however, repeat imaging at 2 years revealed symmetric volume loss of bilateral caudate heads and bodies, with lesser putaminal involvement, without abnormal mineralization or signal intensity. No cerebellar atrophy, hypomyelination, or white matter changes were present apart from nonspecific central tegmental tract signal.

Prior clinical genetic testing was non-diagnostic. A clinical gene panel returned only variants of uncertain significance in TOR1A and KIDINS220, both predicted benign by the testing laboratory. Chromosomal microarray (CMA) identified a small (~0.1 Mb) partial duplication of WDR19 of uncertain clinical significance, with no other pathogenic copy number changes and no extended regions of absence of heterozygosity suggestive of consanguinity or uniparental disomy. Clinical whole-exome sequencing (WES) with mitochondrial genome analysis, performed at 20 months of age, was reported as normal. The VPS16 variant was ultimately identified through research-grade trio short-read whole-genome sequencing performed under the HSPseq program at Boston Children's Hospital (see Genetic Analysis, below).

Comment

This case represents the earliest reported onset of VPS16-associated dystonia. Previously, a review of 81 cases noted a mean onset of 16.5 years, with the youngest prior case reported at 2 years of age.(1, 2) Our patient was initiated on trihexyphenidyl; however, recent data suggests limited efficacy of pharmacotherapy in this condition.(1) Deep brain stimulation (DBS) of the globus pallidus interna currently represents the most effective intervention, with a recent multicenter study of 26 patients demonstrating mean motor improvement of 41.6% and a 73% responder rate at extended follow-up.(3) Future candidacy for DBS in this patient will depend on disease progression. Functional characterization of patient-derived fibroblasts supports the pathogenicity of the p.Ala466Thr variant. Consistent with previous studies of VPS16 and other HOPS-complex disorders, electron microscopy revealed vacuolar abnormalities indicative of impaired autophagosome-lysosome fusion.(4-7) These findings confirm that the missense variant produces cellular defects comparable to loss-of-function alleles.(4)

Genetic Analysis

Trio short-read whole-genome sequencing (WGS) was performed. Variants were filtered based on population frequency (gnomAD v4.1.0) and predicted pathogenicity. No additional pathogenic variants were identified in known dystonia or metabolic disease genes. The VPS16 variant (NM_022575.4:c.1396G>A) was confirmed via Sanger sequencing. In silico algorithms supported pathogenicity (CADD 29.2, REVEL 0.844, AlphaMissense 0.8917). The variant was classified as likely pathogenic based on ACMG criteria, supported by de novo occurrence, consistent phenotype, and absence from population databases.

Fibroblast culture conditions

Primary human skin fibroblast lines were established from individuals. Written informed consent for routine skin punch biopsies was obtained from all participants or their legal guardians. Fibroblasts were derived from the VPS16-deficient patient and from healthy control. Primary fibroblasts were maintained in DMEM high glucose (Gibco, #11960044) supplemented with 20% fetal bovine serum (Gibco, #10082147), and 1% Penicillin-Streptomycin (Gibco, #15140122). Fibroblasts were cultured at 37°C in a humidified incubator with 5% CO_2. For transmission electron microscopy (TEM), fibroblasts were seeded as monolayers onto Aclar coverslips in 24-well plates. For immunoblotting experiments, fibroblasts were seeded onto 6-well plates and processed at comparable confluence across conditions.

Transmission electron microscopy (TEM)

Transmission electron microscopy was performed on fibroblast monolayers grown on Aclar coverslips. Cells were fixed using a fixative consisting of 2.5% glutaraldehyde, 1.25% paraformaldehyde, and 0.03% picric acid in 0.1 M sodium cacodylate buffer (pH 7.4) for 1 hour at room temperature. Following fixation, samples were washed in 0.1 M sodium cacodylate buffer (pH 7.4) and treated for 30 minutes with 1% osmium tetroxide (OsO_4)

and 1.5% potassium ferrocyanide (KFeCN□). Cells were subsequently washed in water, rinsed once in maleate buffer, and incubated in 1% uranyl acetate in maleate buffer for 30 minutes. After additional washes in water, samples were dehydrated through a graded ethanol series (50%, 70%, 95%, and two changes of 100% ethanol, 5 minutes each step). Dehydrated samples were embedded in TAAB Epon resin (TAAB Laboratories Equipment Ltd, # T031) and polymerized at 60°C for 48 hours. Following polymerization, the Aclar coverslips were removed, and small regions (about 1 × 1 mm) of the embedded cell monolayer were excised and remounted onto Epon blocks. Ultrathin sections (around 80 nm) were cut using a Reichert Ultracut-S microtome, collected on copper grids, and counterstained with lead citrate. Sections were examined using a TecnaiG^2 Spirit BioTWIN transmission electron microscope, and images were acquired with an AMT Nanosprint 43-MKII camera.

Immunoblotting

Immunoblotting was performed using standard procedures. Fibroblasts were lysed in RIPA lysis buffer (Thermo Fisher Scientific, #89900) supplemented with Protease and Phosphatase Inhibitor cocktail (Thermo Fisher Scientific, #78440). Protein concentrations were determined using the Pierce BCA Protein Assay Kit (Thermo Fisher Scientific, #23225). Equal amounts of protein were denatured in LDS Sample Buffer (Thermo Fisher Scientific, #NP0007) and Sample Reducing Agent (Thermo Fisher Scientific, #NP0009) boiling at 70°C for 10 minutes. And separated by SDS–PAGE using 4–12% or 12% Bis-Tris gels (Thermo Fisher Scientific, #NP0321BOX or #NP0343BOX) with Running Buffer (Thermo Fisher Scientific, #NP0001). Proteins were transferred onto PVDF membranes (EMD Millipore, #SLHVR33RS) with Transfer Buffer (Thermo Fisher Scientific, #NP00061). Membranes were blocked using Blocking Buffer (LICORbio#927-70001) and incubated overnight at 4°C with the indicated primary antibodies. Near-infrared fluorescent–labeled secondary antibodies were applied, and the bands were detected using the Odyssey imager (LI-COR Biosciences). Band intensities were quantified using ImageJ software. Antibodies used in this study are listed in Supplemental Table 1. Uncropped immunoblots are provided in the Supplemental Figure 2.

Quantification and statistical analysis

Data are presented as mean ± SEM from three independent biological replicates. Statistical comparisons between control and VPS16-deficient samples were performed using two-tailed unpaired t-test. TEM count data were analyzed using a two-tailed Mann–Whitney test. Differences were considered significant at $P < 0.05$.

Supplemental Figure 1. *VPS16 conservation and TEM analysis.*

(A) Multiple sequence alignment of VPS16 across species. The residue affected by the patient missense variant is boxed in red and is conserved across species.

(B) Additional transmission electron microscopy images of VPS16 p.Ala466Thr fibroblasts. Autolysosome-like structures are highlighted by dashed yellow circles.

(C) Semi-quantitative analysis of autolysosome-like structures per nuclear-containing cell profile. Approximately 10 independent cell profiles per condition were analyzed. Data were analyzed using a two-tailed Mann–Whitney test.

Supplemental Figure 2. *Uncropped immunoblots.*
Uncropped Western blot images corresponding to the data shown in Figure 1E.

Supplemental Video 1. *Examination of the patient at age 2 years: The patient demonstrates appendicular hypertonia with prominent dystonic posturing. Key features include sustained extension and plantarflexion of the lower extremities, accompanied by bilateral fisting of the hands.*

Sex as a biological variable

This is a report of a single female individual. Sex as a biological variable is not assessed.

Study approval

This study was conducted in accordance with the Declaration of Helsinki and approved by the Institutional Review Board of Boston Children's Hospital (IRB protocol IRB-P00039630). Written informed consent was obtained from the patient's parent/legal guardian prior to enrollment, including consent for publication of clinical information, imaging data, and video recordings.

Data availability

Data are available from the corresponding author upon reasonable request.

Author contributions

Enrique Gonzalez Saez-Diez: Conceptualization, Methodology, Investigation, Formal analysis, Data visualization, Writing – original draft, Writing – review & editing.
Xutong Xue: Conceptualization, Methodology, Investigation (cellular experiments: TEM, immunoblotting, fibroblast culture), Formal analysis, Data visualization, Writing – original draft, Writing – review & editing.
Amy Tam: Investigation, Formal analysis, Writing – review & editing.
Hyo-Min Kim: Investigation, Formal analysis, Writing – review & editing.
Siofra Carty: Investigation, Writing – review & editing.
Joshua Rong: Investigation, Writing – review & editing.
Monica Ferrer-Socorro: Investigation, Writing – review & editing.
Kathryn Yang: Investigation, Writing – review & editing.
Darius Ebrahimi-Fakhari: Conceptualization, Supervision, Resources, Funding acquisition, Writing – original draft, Writing – review & editing, Project administration.

Acknowledgments

The authors thank their patient and her family for supporting this research project. This study was supported by the Children's Rare Disease Collaborative (CRDC) at Boston Children's Hospital. Research in the Ebrahimi-Fakhari Laboratory is supported by the National Institute of Neurological Disorders and Stroke (K08NS123552, 1U54NS148312), the Spastic Paraplegia Foundation, the Dystonia Medical Research Foundation, the Boston Children's Hospital Translational Research Program, the Boston Children's Hospital TIDO Accelerator Award. M.F.S. received a fellowship from the Dystonia Medical Research Foundation.

Funding sources

Research in the Ebrahimi-Fakhari Laboratory is supported by the National Institute of Neurological Disorders and Stroke (K08NS123552, 1U54NS148312), the Spastic Paraplegia Foundation, the Dystonia Medical Research Foundation, the Boston Children's Hospital Translational Research Program, the Boston Children's Hospital TIDO Accelerator Award. M.F.S. received a fellowship from the Dystonia Medical Research Foundation.

Supplemental references

1. *Panigrahi B, et al. VPS16-Related Dystonia: Expanding the Clinical Spectrum and Therapeutic Insights. Mov Disord Clin Pract. 2025.*
2. *Mahale RR, and Padmanabha H. VPS16-Associated Dystonia: A Cohort-Based Clinical, Imaging and Genetic Profile. Tremor and Other Hyperkinetic Movements. 2025;15(1).*
3. *Svorenova T, et al. Deep Brain Stimulation for VPS16-Related Dystonia: A Multicenter Study. Ann Neurol. 2025;98(4):711-25.*
4. *Steel D, et al. Loss-of-Function Variants in HOPS Complex Genes VPS16 and VPS41 Cause Early Onset Dystonia Associated with Lysosomal Abnormalities. Annals of Neurology. 2020;88(5):867-77.*
5. *Monfrini E, et al. A Novel Homozygous VPS11 Variant May Cause Generalized Dystonia. Ann Neurol. 2021;89(4):834-9.*
6. *van der Beek J, et al. Loss of the HOPS complex disrupts early-to-late endosome transition, impairs endosomal recycling and induces accumulation of amphisomes. Mol Biol Cell. 2024;35(3):ar40.*
7. *van der Welle REN, et al. Neurodegenerative VPS41 variants inhibit HOPS function and mTORC1-dependent TFEB/TFE3 regulation. EMBO Molecular Medicine. 2021;13(5):e13258.*
