# Supplementary material for: De novo VPS16 missense variant causes infantile-onset dystonia with defective autophagic flux
Source: JCI Insight. 2026 Jun 30;11(16):e207998. doi: 10.1172/jci.insight.207998 (PMC13505382; doi:10.1172/jci.insight.207998)
Supplement: Supplementary file 2 [file jciinsight-11-207998-s020.pdf]

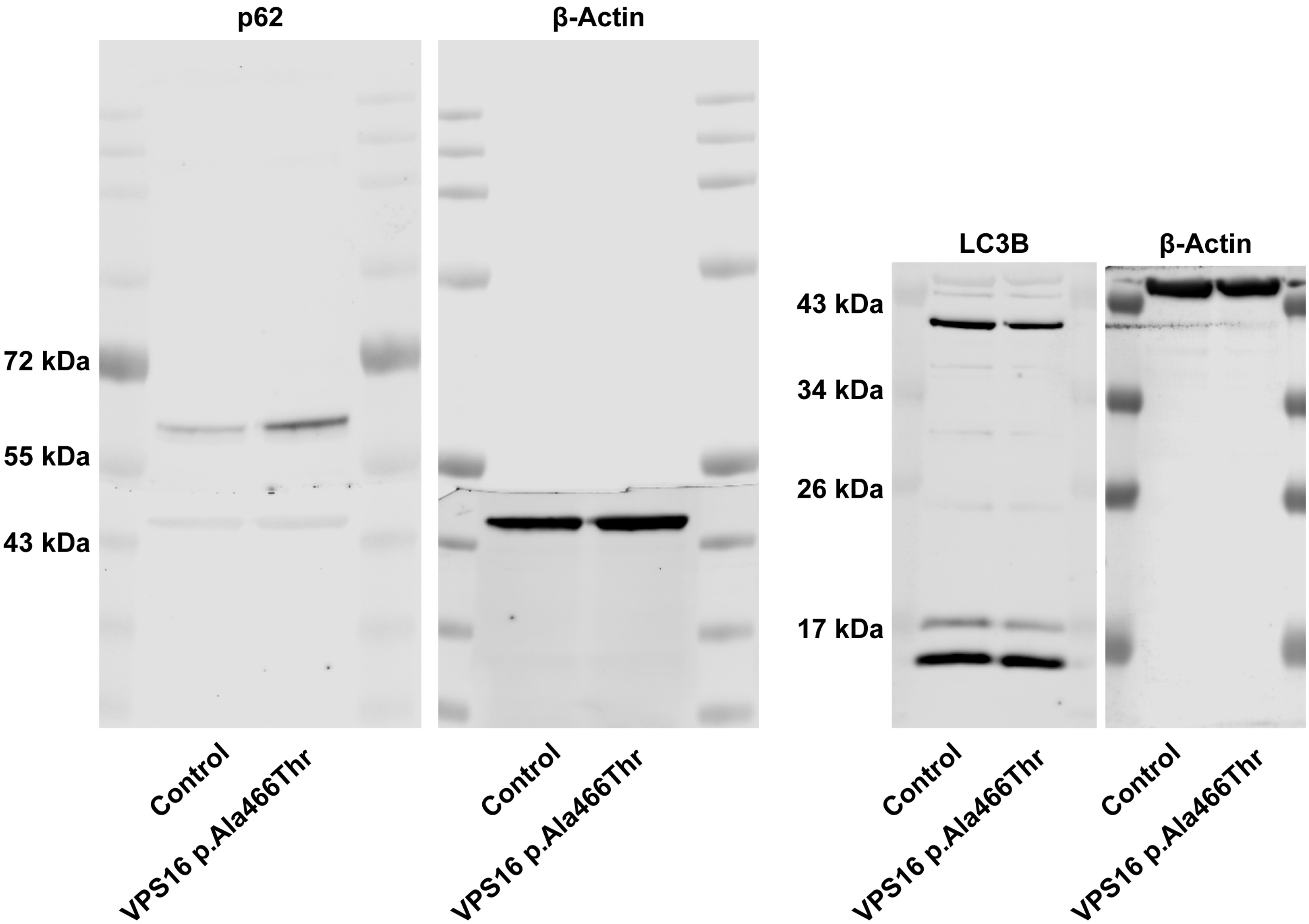
p62
β-Actin
LC3B
β-Actin
72 kDa
55 kDa
43 kDa
43 kDa
34 kDa
26 kDa
17 kDa
Control
VPS16 p.Ala466Thr
Control
VPS16 p.Ala466Thr
Control
VPS16 p.Ala466Thr
Control
VPS16 p.Ala466Thr
